# Genetic diversity and population structure of *Amorphophallus albus*, a plant species with extremely small populations (PSESP) endemic to dry-hot valley of Jinsha River

**DOI:** 10.1186/s12863-020-00910-x

**Published:** 2020-09-12

**Authors:** Rong Tang, Erxi Liu, Yazhou Zhang, Johann Schinnerl, Weibang Sun, Gao Chen

**Affiliations:** 1 Yunnan Key Laboratory for Integrative Conservation of Plant Species with Extremely Small Populations, Kunming Institute of Botany, Chinese Academy of Sciences, Kunming, 650201 China; 2 CAS Key Laboratory for Plant Biodiversity and Biogeography of East Asia, Kunming Institute of Botany, Chinese Academy of Sciences, Kunming, 650201 China; 3 University of Chinese Academy of Science, Beijing, 100049 China; 4 Enshi Autonomous Prefecture Academy of Agricultural Sciences, Enshi, 445000 China; 5 Department of Botany and Biodiversity Research, University of Vienna, Vienna, Austria

**Keywords:** *Amorphophallus albus*, Genetic diversity, Population structure, Conservation, PSESP

## Abstract

**Background:**

*Amorphophallus albus* P. Y. Liu & J. F. Chen (Araceae) is a plant species with extremely small populations (PSESP) and an important economic crop endemic to dry-hot valleys along the Jinsha River. In order to gain information for sustaining the development and conservation of *A. albus*, we studied the genetic diversity and population structure of this species using microsatellite markers (SSR). In this study, we analysed 364 individuals belonging to 24 populations, including four wild populations and three ex-situ cultivated populations, collected in the provinces Yunnan, Sichuan and Hubei.

**Results:**

The population genetic analyses indicated that *A. albus* possesses moderate genetic diversity with the percentage of polymorphic loci (*PPL*) from 69.23 to 100%, an expected heterozygosity (*He*) of 0.504 and an average Shannon’s Information Index (*I*) 0.912. Analysis of molecular variance (AMOVA) indicated that most of the variance (71%) resided within populations and the estimated gene flow (*Nm*) was 0.61. The results of UPGMA cluster tree, STRUCTURE analyses together with the Mantel test (R^2^ = 0.352, *P* < 0.01) indicated that geographically closely located populations are clustered together with some exceptions.

**Conclusions:**

Our results showed that *A. albus* still possesses moderate genetic variation in most of the studied populations, and for now, most cultivated populations were naturally distributed but still some reintroduction exists. For sustaining the present genetic variation, some protections measures are necessary for the wild populations and also for the cultivated ones with high genetic diversity.

## Background

*Amorphophallus albus* P. Y. Liu & J. F. Chen (Araceae) is a herbaceous perennial plant species occurring along the Jinsha River in southern Sichuan and northern Yunnan. It is growing in open forests between 800 to 1000 m altitude on arid locations [1]. It’s an economic crop widely used for food, medicine and industry due to the glucomannan (KGM) content in its tubers [2, 3]. The high quality and purity of KGM obtained from *A. albus* makes this species the second most cultivated *Amophophallus* species after *A. konjac* K. Koch in China [4]. At present, the cultivation of *A. albus* is one of the pillars in agriculture of counties along the Jinsha River. For example, in Jinyang, the cultivation area is more than 3333 ha with commodity production more than 30,000 kg and a production value of about 120 million Yuan every year [5]. Moreover, the resistance against high temperatures and drought tolerance of *A. albus* are important factors for the breeding of drought-resistant varieties [6]. Since it has been cultivated for hundreds of years, wild populations are almost disappeared. In 2017, *A. albus* was listed as a potential targeted PSESP (Plant Species with Extremely Small Populations) for the China National Key Program of Survey and Germplasm Conservation of Plant Species with Extremely Small Populations in Southwest China [7].

Genetic diversity is the variation of the genetic material of organisms and the basis for adaptation of species to the natural environment [8]. Characteristics as such provides many useful information about history, adaptive potentials and relationships, and is also basis for phylogeny or classification of taxa [9, 10]. Analyses of molecular markers, especially microsatellites, are widely applied to reveal genetic diversity of threatened species in recent years [11–13]. Endangered plant species usually have low genetic variation, like *Abies ziyuanensis* L.K. Fu & S.L. Mo (*He* = 0.337) [14], *Elaeagnus mollis* Diels (*He* = 0.2683, *I* = 0.3815) [15, 16]. According to Nybom [17], the average expected heterozygosity (*He*) of endemic plant species analyzed by microsatellite is 0.42, whilst for species with narrow distribution is 0.56 and 0.62 for widespread species, respectively. At present, studies focusing on genetic diversity of Araceae species were valued mostly by the first generation of molecular markers including RFLP [18], RAPD [19, 20], AFLP [21–23], only *Amorphophallus paeoniifolius* (Dennst.) Nicolson and *Xanthosoma sagittifolium* (L.) Schott were analysed by microsatellites [24, 25], and inter-simple sequence repeat (ISSR) markers [26–28]. Among these molecular markers, microsatellite markers have high mutation rate, large amount of information, large numbers of loci, and low requirements for DNA quantity/purity. Thus, they play an important role in genetic diversity of plant species [29].

In the present study, we used 13 pairs of microsatellite loci to analyse the genetic diversity and population structure of *A. albus* from 24 populations including four wild populations and 17 in-situ cultivated populations as well as three ex-situ populations for following purposes: 1) to explore the trends of natural formation and evolution; 2) to provide a theoretical basis for conservation; 3) to reveal the net of introduction into the present cultivation area and 4) to determine the origin of this species*.*

## Results

### Genetic diversity

In this study, we finally collected 364 individuals from 24 populations including four wild populations and 17 in-situ populations together with three ex-situ populations, each populations’ information are listed in Table 1. In addition, 13 pairs of microsatellite primers are screened to analyse genetic diversity and population structure of *A. albus* (Table 2). The genetic characters of 13 microsatellite loci are listed in Table 3, according to the results, the polymorphism information content (*PIC*) range from 0.439 to 0.869 with an average of 0.683, which indicate a high polymorphism and their suitability for genetic analysis, the null allele frequency range from 0.013 to 0.523 with an average of 0.195, meanwhile, only three loci (TR6, TR17, TR54) were detected with null alleles through Micro-checker software [30]. In total, 100 alleles were detected, each locus had 3–13 alleles with an average of 8.7 alleles per locus. The genetic diversity parameters assessed by these microsatellite primers are listed in Table 4. Briefly, the average allele number (*Na*) was 3.619, with a range from 1.846 (SDC/TWC) to 4.615 (HLX). The average effective allele number (*Ne*) was 2.372, with a range from 1.541 (SLC) to 3.404 (LIZ). The average Shannon’s Information Index (*I*), observed heterozygosity (*Ho*), expected heterozygosity (*He*) are 0.912, 0.528, 0.504 on average, respectively. The percentage of polymorphic loci (*PPL*) of each population ranged from 69.23 to 100%. Based on the results, population LIZ (*He* = 0.667, *I* = 1.245) and HLX (*He* = 0.654, *I* = 1.238) showed the highest genetic diversity, while population SLC (*He* = 0.293, *I* = 0.511) and SDC (*He* = 0.334, *I* = 0.494) showed the lowest genetic diversity.

**Table 1:** Location and sampling site characteristics for all *Amorphophallus albus* populations in the present study

Location	Pop.	Longitude	Latitude	Altitude (m)	Sample size	Habitat
Jingyang County, Sichuan, China	SJX	102°56′54.39″E	27°25′5.39″N	588	17	Wild
	TPX	103°13′22.07″E	27°39′14.36 ″N	783	17	Wild
	LGLH	103°10′10.93″E	27°34′26.57″N	826	9	Wild
	TSC	103°10′2.38″E	27°34′47.52″N	1023	16	Wild
	MYZ	103°16′30.72″E	27°41′7.73″N	1788	17	Cultivation
	HLX	103°8′10.56″E	27°29′43.48″N	1102	17	Cultivation
Leibo County, Sichuan, China	YCC	103°47′49.42″E	28°29′48.81″N	625	17	Cultivation
	QJW	103°25′37.28″E	28°1′31.64″N	916	16	Cultivation
Pingshan County, Sichuan, China	JLC	103°48′23.37″E	28°49′45.35″N	775	17	Cultivation
	SLC	103°59′54.13″E	28°38′15.82″N	885	17	Cultivation
	TWC	103°42′34.82″E	28°38′2.74″N	774	4	Cultivation
Yongshan County, Yunnan, China	ML	103°16′25.40″E	27°33′8.56″N	1323	17	Cultivation
	HH	103°31′08.81″E	28°0′20.62″N	1117	15	Cultivation
	BJC	103°55′59.31″E	28°20′19.04″N	798	14	Cultivation
	BJ	103°31′16.30″E	28°7′27.81″N	1254	14	Cultivation
	SYC	103°36′7.56″E	28°17′25.85″N	1422	17	Cultivation
	STC	103°47′8.56″E	28°13′59.40″N	818	15	Cultivation
	LIZ	103°28′26.79″E	27°44′56.99″N	1302	15	Cultivation
	XP	103°31′45.10″E	27°52′9.64″N	1204	15	Cultivation
Zhaoyang District, Yunnan, China	TBC	103°10′36.72″E	27°24′21.72″N	1707	17	Cultivation
Suijiang County, Yunnan, China	SDC	104°8′19.35″E	28°32′49.71″N	807	15	Cultivation
Enshi Prefecture, Hubei, China	HB	109°28′34.19″E	30°19′4.52″N	425	15	Ex-situ cult
Fuyuan County, Yunnan, China	FY	104°17′35.38″E	25°22′56.10″N	1795	17	Ex-situ cult
Panlong District, Yunnan China	KIB	102°44′37.51″E	25°8′11.40″N	1936	14	Ex-situ cult

*Pop*, Population

**Table 2:** Detailed information of 13 microsatellite loci in *Amorphophallus albus*

Locus	Repeat	Ranges of allele sizes	Tm(℃)	Primer sequence (5′ → 3′)
**TR6**	(CT)7	126–152	55.4	GCCCCATGTCTCACCTGTAT
				TATGCACATGGCAAAGCCTA
**TR7**	(CT)7	202–228	55.4	ATTGGAGCAGAATTTGTGGG
				CCCCTCTCTGTGAAGAACCA
**TR8**	(CT)7	116–128	55.4	TGAACTTGTCTTCTCCCGCT
				ATCGAGGGAGCAATTAGGGT
**TR9**	(CT)7	143–163	55.4	GGGATTGGAAGAGGAAAGGA
				CATCAGACACCATCGCAAAC
**TR17**	(GA)10	133–168	58.5	GAGGAACGGTGGTCACTCAT
				CTCTCCCCTCTCTGTTTCGC
**TR26**	(GA)6	286–318	53.4	TTGATGATTTTTCTGCCGGT
				TGATTGCTGTCTACCCGACA
**TR34**	(TC)10	208–232	54.4	TGGTGCAAAACAAGGTGGTA
				AATGTGCGACCCACACTACA
**TR39**	(TC)15	201–227	55.4	GTTGCTGGTAACGAGAAGGC
				TTCAGGGAAAACCGAGAGAA
**TR49**	(TC)7	275–315	58.5	GCTGCTACCAAGTGAGGAGG
				CCGAACCTTGTTAGCTGAGG
**TR52**	(TC)8	135–171	57.4	ACAAACTCCACTGCCTGTCC
				CTGCCAAGTGATGACCAGTG
**TR54**	(TC)9	130–156	54.4	CGTTTTGATTTGATTCACCG
				CGACTCAGACGTGCCGTATT
**TR68**	(GCT)8	131–202	55.4	GCAAAATCCCAGACCACACT
				CGAAAGTTCTGCCAAGGAAC
**TR69**	(GGA)6	136–202	58.5	GAGCTCGAACCTGCCTACTG
				TACACTACCGATGCTGTCGC

Tm, annealing temperature

**Table 3:** Polymorphism parameters and *F*-statistics of 13 microsatellite loci in *Amorphophallus albus*

Locus	***Ho***	***He***	***F (null)***	***PIC***	***Fis***	***Fit***	***Fst***	***Nm***	***HW***
**TR6**	0.439	0.712	0.250	0.690	−0.001	0.344	0.345	0.475	***
**TR7**	0.601	0.695	0.067	0.643	−0.157	0.146	0.262	0.706	***
**TR8**	0.632	0.650	0.013	0.575	−0.365	−0.064	0.221	0.882	NS
**TR9**	0.543	0.768	0.175	0.733	−0.113	0.258	0.333	0.500	***
**TR17**	0.566	0.833	0.191	0.811	−0.036	0.260	0.286	0.623	***
**TR26**	0.263	0.484	0.324	0.439	0.139	0.455	0.366	0.433	***
**TR34**	0.667	0.752	0.061	0.728	−0.170	0.127	0.254	0.736	NS
**TR39**	0.535	0.761	0.178	0.725	−0.076	0.315	0.364	0.437	***
**TR49**	0.716	0.793	0.042	0.772	−0.194	0.085	0.234	0.819	***
**TR52**	0.416	0.882	0.362	0.869	0.273	0.531	0.355	0.455	***
**TR54**	0.428	0.648	0.203	0.592	0.312	0.601	0.419	0.346	***
**TR68**	0.470	0.623	0.148	0.574	−0.043	0.367	0.394	0.385	***
**TR69**	0.242	0.720	0.523	0.732	−0.088	0.284	0.342	0.481	***
**Mean**	0.501	0.321	0.195	0.683	−0.040	0.285	0.321	0.560	

*Ho*, observed heterozygosity; *He*, expected heterozygosity; *F (null)*, null allele frequency; *PIC*, polymorphism information content; *Fis*, mean inbreeding coefficient within individuals relative to subpopulation; *Fit*, mean inbreeding coefficient within individuals relative to the total population; *Fst*, mean inbreeding coefficient within subpopulation relative to the total population; *Nm*, gene flow; *HW*: Hardy-Weinberg equilibrium. ***, *P* < 0.001; NS: not significant

**Table 4:** Genetic characters of 24 *Amorphophallus albus* populations based on 13 microsatellite loci

Pop	***N***	***Na***	***Ne***	***I***	***Ho***	***He***	***PPL***
**BJ**	13.462	3.385	2.062	0.860	0.421	0.480	100.00%
**BJC**	13.923	3.615	2.311	0.864	0.437	0.476	100.00%
**FY**	17.000	3.538	2.568	0.983	0.557	0.545	100.00%
**HB**	14.923	4.077	2.144	0.917	0.509	0.487	100.00%
**HH**	14.538	4.000	2.974	1.100	0.579	0.594	92.31%
**HLX**	16.538	4.615	3.233	1.238	0.601	0.654	100.00%
**JLC**	16.923	2.462	1.762	0.604	0.548	0.369	92.31%
**KIB**	13.923	4.231	2.068	0.845	0.354	0.422	100.00%
**LGLH**	9.000	2.308	1.714	0.534	0.530	0.330	76.92%
**ML**	16.692	3.923	2.600	1.042	0.641	0.573	100.00%
**MYZ**	16.769	4.462	2.025	0.877	0.481	0.457	100.00%
**QJW**	15.923	4.231	2.644	1.082	0.539	0.591	100.00%
**SJX**	16.385	3.923	2.449	0.994	0.438	0.551	100.00%
**SLC**	16.923	2.769	1.541	0.511	0.362	0.293	84.62%
**SYC**	17.000	4.308	2.657	1.100	0.502	0.583	100.00%
**TBC**	16.923	4.154	2.558	1.041	0.436	0.553	100.00%
**TPX**	16.615	4.231	2.601	1.037	0.573	0.547	100.00%
**TSC**	15.846	3.615	2.592	1.014	0.470	0.551	92.31%
**TWC**	4.000	1.846	1.815	0.576	0.769	0.413	84.62%
**YCC**	17.000	3.462	2.397	0.898	0.457	0.496	92.31%
**LIZ**	14.462	3.308	3.404	1.245	0.645	0.667	100.00%
**SDC**	15.000	1.846	1.749	0.494	0.615	0.334	69.23%
**STC**	14.923	3.385	2.358	0.919	0.556	0.525	100.00%
**XP**	14.615	4.154	2.712	1.105	0.647	0.598	100.00%
**Mean**	14.971	3.619	2.372	0.912	0.528	0.504	95.19%

*N*, sample size; *Na*, observed allele number; *Ne*, effective allele number; *I*, Shannon’s information index; *He*, expected heterozygosity; *Ho*, observed heterozygosity; *F*, fixation index; *PPL*, percentage of polymorphic loci

### Genetic differentiation

According to the results of AMOVA analysis, about 29.23% of the total genetic variation occurred among populations, whereas the remaining 70.77% of the variation occurred within populations (Table 5). The estimated population differentiation coefficient (*Fst*) and estimated gene flow (*Nm*) was 0.29 and 0.61, respectively. The results of *F*-statistics in each locus are shown in Table 3. The results indicated that the inbreeding coefficients (*Fis*) of most loci were less than zero with an average of − 0.04. The estimated population differentiation coefficient (*Fst*) of each locus ranged from 0.221 to 0.419, with an average of 0.321, the average gene flow (*Nm*) of all the loci was 0.560, almost identical to the results calculated by AMOVA.

**Table 5:** Analysis of molecular variance (AMOVA) of genetic diversity in *Amorphophallus albus*

Source of variation	Degree of freedom	Total variance	Variation component	Percentage of variation
**Among population**	23	968.88	1.29	29.23%
**Within population**	704	2195.60	3.12	70.77%
**Total**	727	3164.47	4.41	100.00%

### Population structure

**Fig. 1:**
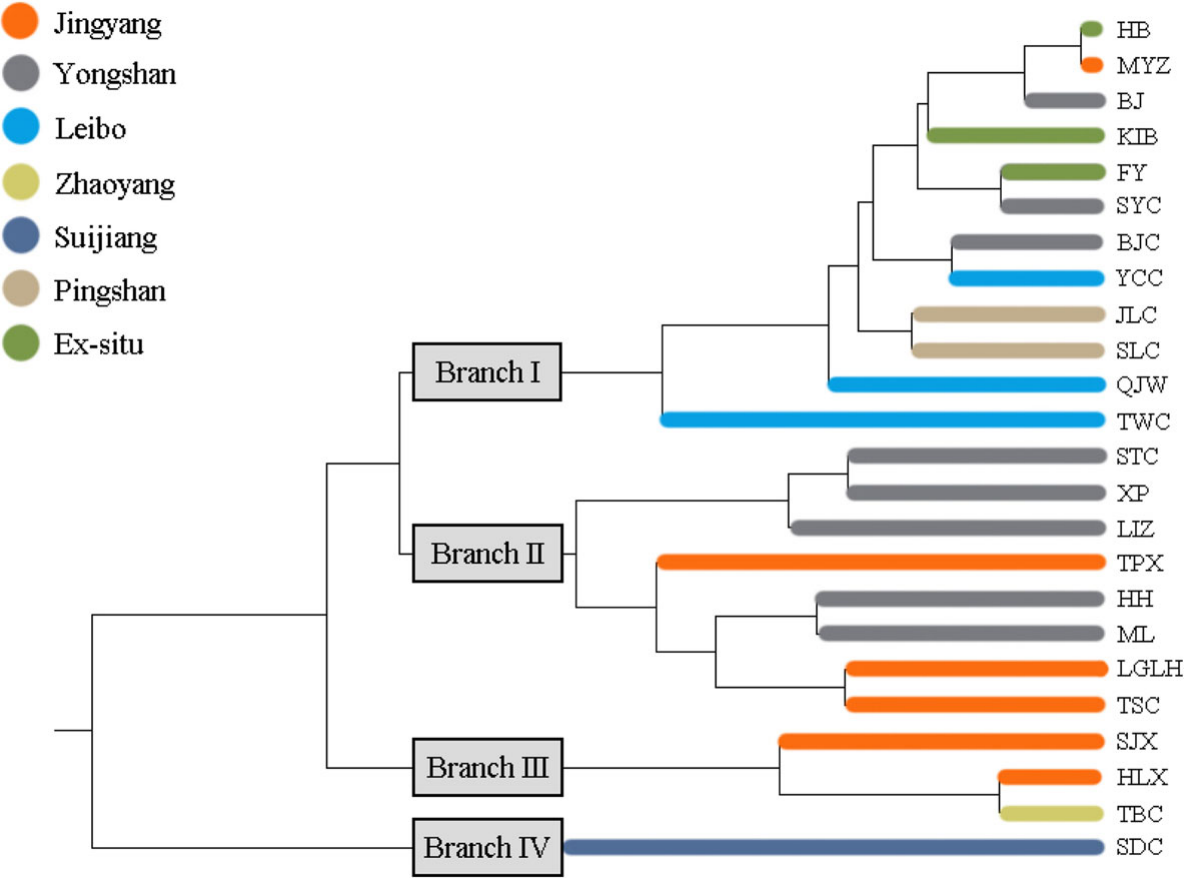
Dendrogram based on *Nei*’s genetic distance of *Amorphophallus albus.* Colors represent different regions

**Fig. 2:**
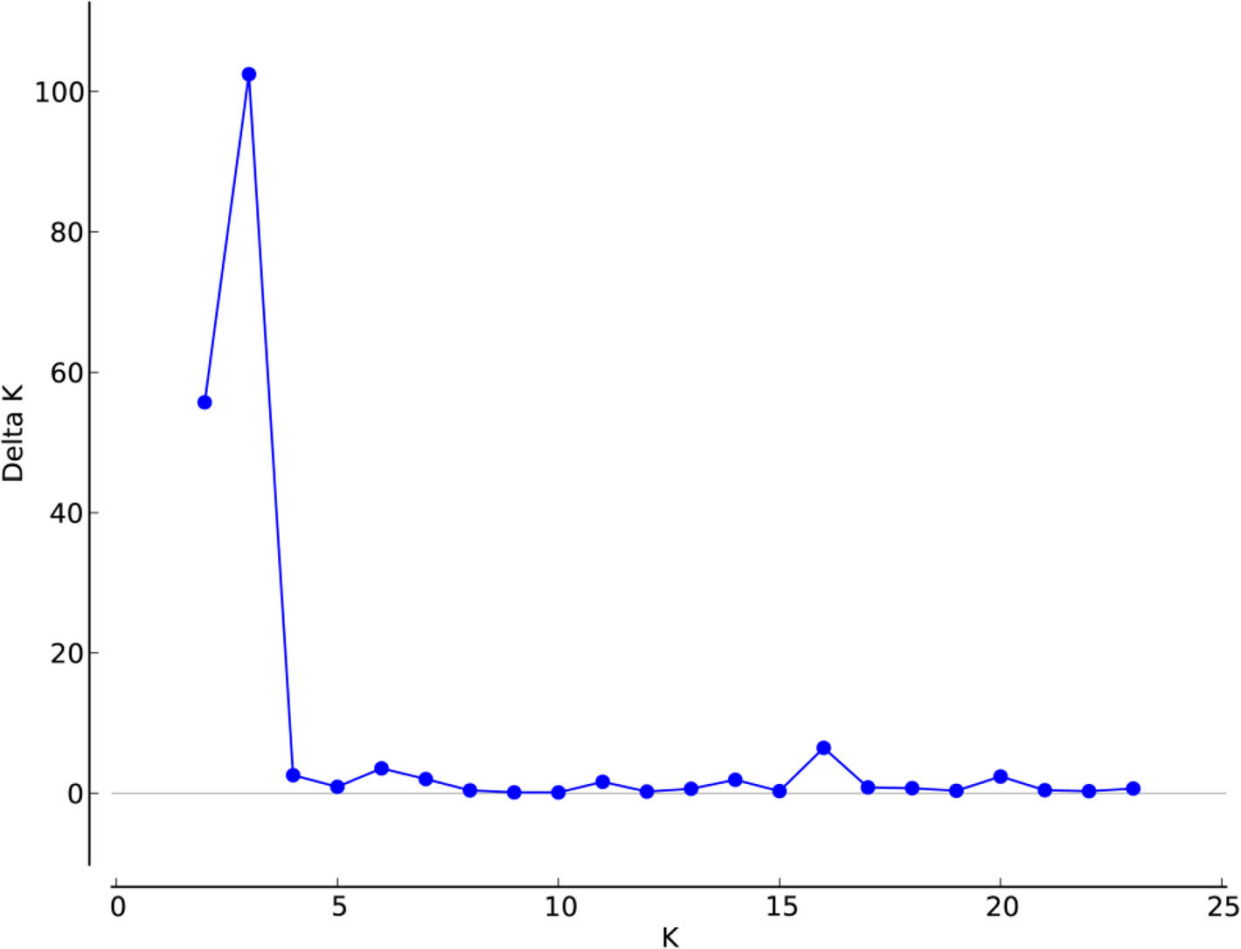
Graph showing the relationship between ΔK and K

**Fig. 3:**
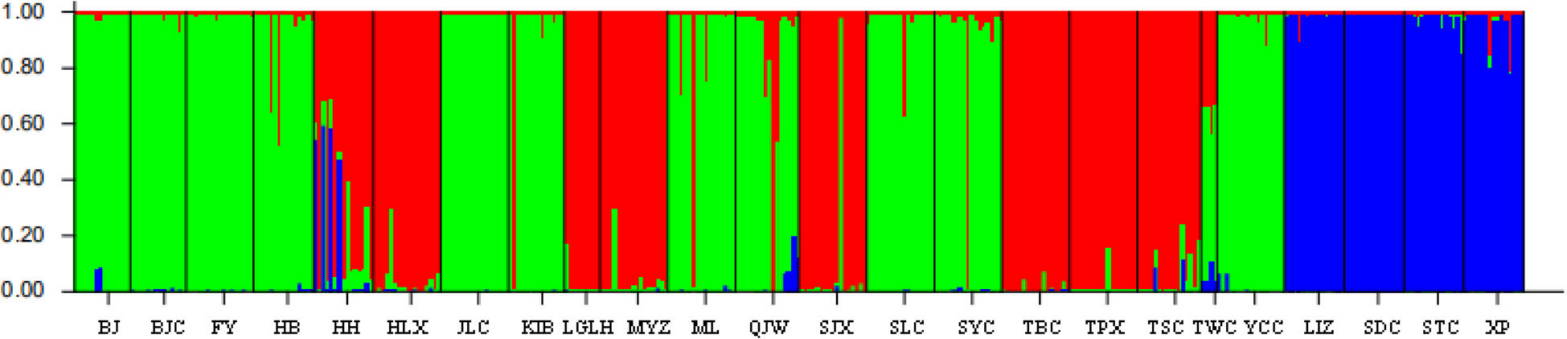
Structure dendrogram in clustering analysis among 24 populations of *Amorphophallus albus*

**Fig. 4:**
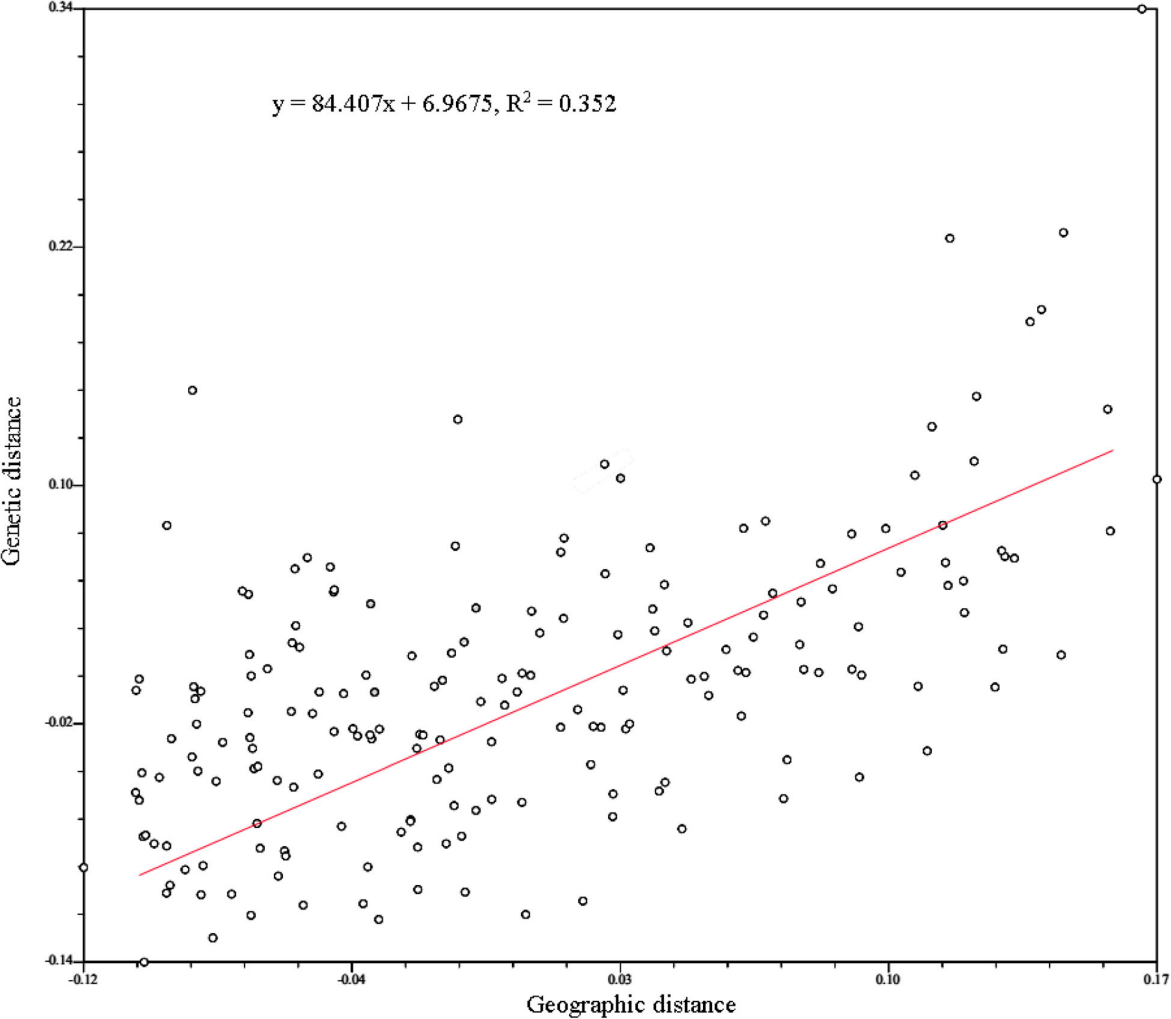
Mantel test for correlation of genetic and geographic distances in *Amorphophallus albus*

The genetic identities (above diagonal) and genetic distances (below diagonal) of population pairs were listed in Table S1. Among all the populations, the farthest genetic distance and lowest genetic identity existed in SJX and SDC, while MYZ and HB had the nearest genetic distance and highest genetic identity. The dendrogram based on *Nei*’s genetic distance (Fig. 1) showed that all the populations were clustered in four groups where geographically contiguous populations were more genetically related than distant populations. Specifically, the population SDC alone gathered into IV branch, two populations of Jinyang County (SJX, HLX) and a population of Zhaoyang District (TBC) in the south clustered into III branch, while three populations of Jinyang County (TSC, LGLH, SJX) together with five populations of Yongshan County (ML, HH, LIZ, XP, STC) in central part clustered into II branch. Moreover, the remaining populations from Leibo County, Pingshan County, part of Yongshan County in the north and other three ex-situ cultivated populations gathered into I branch. The Bayesian cluster analysis based on the STRUCTURE software run K from 1 to 24, according to the evaluation criteria and calculation formula of Evanno [31], the relationship of ΔK and K are shown in Fig. 2, the results indicated that ΔK reached the peak when K = 3. Thus, the populations were clustered into three branches (Fig. 3) by Bayesian cluster analysis. Among them, six populations from Jinyang County, one population from Yongshan and one population from Zhaoyang District clustered together. Four populations including three from Yongshan County and one from Suijiang County clustered together, the remaining population formed the biggest branch which includes populations from Yongshan County, Leibo County, Pingshan County and ex-situ cultivation. Lastly, the Mantel test showed that the population genetic distance was positively correlated with geographic distance (R^2^ = 0.352, Fig. 4).

## Discussion

In this study, 13 microsatellite loci were analyzed to reveal the genetic diversity and population structure of *A. albus* from 24 populations in Sichuan, Yunnan, Hubei Province and they all expressed high polymorphism with an average *PPL* of 95.19%. According to the results, we observed a moderate genetic diversity of this species (*He* = 0.504, *I* = 0.912). In comparison, the genetic diversity observed was lower than in other studied *Amorphophallus* species using microsatellite markers, e.g., in *A. paeoniifolius* (*He* = 0.598, *I* = 1.172) [32], but higher than the estimated mean of genetic diversity of endemic species (*He* = 0.42) summarized by Nybom [17]. Genetic diversity of plant species usually depends on their breeding system, distribution or life form [33, 34]. Generally, perennial species with wide distribution, self-incompatible mating system and seed dispersal by animals possess higher genetic diversity [35]. For *A. albus*, which is a perennial herb with limited distribution showing self-incompatible mating system and endozoochory, it is supposed to have relatively higher genetic diversity. However, as an important economic crop, *A. albus* was inevitably disturbed by human activities such as habitat destruction and over excavation in recent years similar to *A. konjac* [23]. Consequently, wild populations of *A. albus* can hardly be found in nature. Moreover, most farmers, who cultivated this species for commercial purposes, tend to use asexual reproduction to get more corms and shorter life cycles [36]. This finally led to a reduced genetic diversity which is clearly observable in the populations of SDC and JLC. In contrast, some cultivated populations still maintain high genetic diversity, even higher than those wild populations, like HLX and LIZ. Presumably, these populations were transplanted from their native habitats and cultivated without or just little human disturbances. Wild populations comprises of not more than 50 individuals may lose genetic diversity in bottleneck events. Another possible reason is, that the existed wild populations were feral from cultivated populations and did not possess much genetic variation originally. According to our results, the populations with high genetic diversity are almost in or around Jinyang County, whilst the populations with the lowest genetic diversity are present in Pingshan and Suijiang. Based on our results, we assume that Jinyang is the natural origin of *A. albus*, and the gene flow from Jingyang to Pingshan showed a trend of expanding towards east along the river. This pattern could also be observed from other species native to the dry and hot valleys along the Jinsha River [37, 38].

The genetic analysis of *A. albus* indicated a high level of differentiation (*Fst* = 0.29) and low gene flow (*Nm* = 0.61) among populations. According to Wright [39], populations show high genetic differentiation and low gene flow when *Fst* > 0.25/*Nm* < 1. High genetic differentiation may result from heterogeneous environments [40]. Though all the populations distributed along Jinsha River, much differences in temperature, humidity, vegetation form existed between the hot-dry valleys and warm-dry valleys [41]. Additionally, Araceae species commonly pollinated by small insects such as ants, beetles and hover flies [42, 43], and *A. albus* is pollinated by rove beetles (Tang et al., unpublished data). This small insect pollination mating system and the complex geography may have limited gene flow among populations and therefore promoted genetic differentiation of this species [44]. Moreover, though the fruits of *A. albus* possess traits for seed dispersal by birds, but this could not be observed.

The observed fixation coefficient (*Fis*) in most loci were less than zero (Table 3) which indicates a great excess of heterozygosity in this species. This is a common phenomenon resulting from the applied sampling strategy, asexual reproduction, heterosis and too small breeding populations [45–47]. Regarding *A. albus*, sampling may be one of the reasons because quite a number of sampled populations belonged to small populations of less than 50 individuals. Another important reason is asexual reproduction independent whether the plants are cultivated or growing the wild. During cultivation, the farmers usually cut inflorescences in order to get bigger tubers, meanwhile, asexual reproduction allows to harvest commercial konjak faster [23]. In latter case, there are always many ramets around an adult plant, which also could be observed from the related species *A. paeoniifolius* [32]. As a result, asexual reproduction seems to be the main reason for excess of heterozygosity in *A. albus.*

**Fig. 5:**
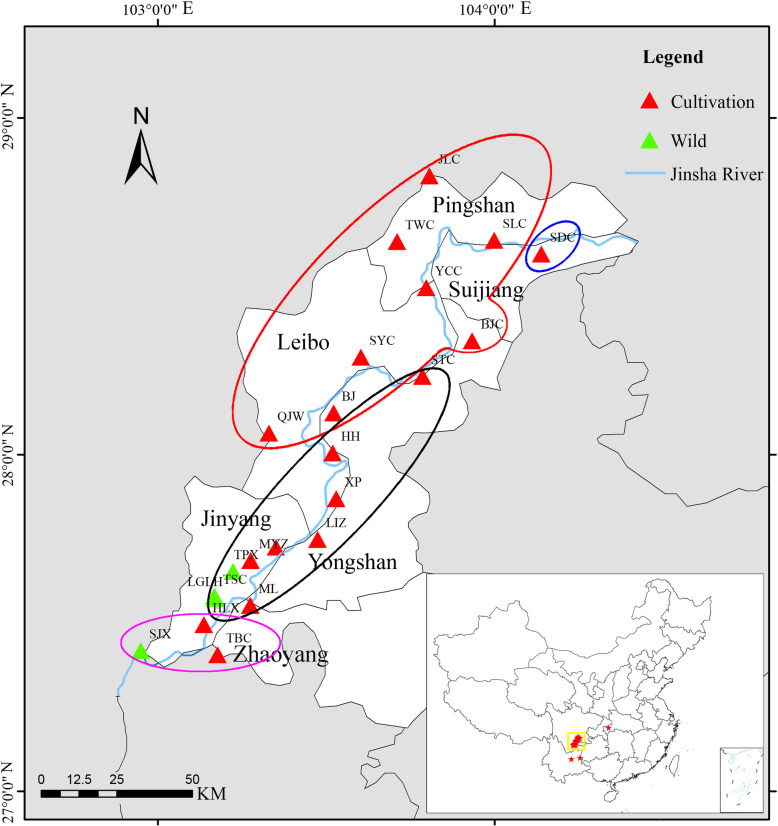
Geographical distribution of the sampled populations of *Amorphophallus albus* along the Jinsha River. Details of each location are given in Table 1

In this study, the results of UPGMA cluster tree, Bayesian cluster analysis and Mantel test indicated that the genetic distance was slightly positive correlated with the geographical distance, and geographically close populations are usually clustered together (Figs. 1 and 5). These results showed that most of the cultivated populations nowadays are collected from native populations. But some populations were put in different places between the two clusters analysis like MYZ. Those populations mostly are the important base of their county of *A. albus* cultivation, every year people buy corms from other counties to increase their own variety. On account of different algorithms of the two software, these populations may be treated differently. Thus, reintroduction was proved to exist in many populations. In addition, the occurrence of three ex-situ cultivated populations in cluster I together with populations of MYZ, BJ and SYC indicated an introduction of these populations either from Yongshan, Leibo or Pingshan County. Reintroduction of plants from MYZ in downstream areas is also conceivable. The occurrence of population SDC in cluster IV (Fig. 1) is may be caused by introgression after hybridization with *A. konjac*. Spatial proximity to the distribution area of the latter species together with the already proved cross-breeding of both species [6] support this assumption.

## Conclusion

In conclusion, most populations of *A. albus* showed moderate genetic diversity due to short domestication history and weak artificial selection. Some of the studied populations showed a fairly low genetic diversity which may resulted from asexual reproduction or bottleneck effects. At present, most populations from the second branch still possess comparatively higher genetic diversity and therefore it is supposed that these populations are the center of genetic diversity of this species*.* Based on our results, we demand the three wild populations and the four cultivation populations of HH, HLX, XP and LIZ as conservation units to sustain most of the genetic variety of *A. albus.* As a next step, ex-situ conservation should also be undertaken in case of ongoing habitat destruction due to human activities. To ensure the genetic diversity, the sexual reproduction of this species must be promoted. These measures would counteract against degradation of this plant species.

## Methods

### Plant collection

Twenty-four populations of *A. albus* samples were collected in the dry-hot valleys along the Jinsha River in the provinces Yunnan and Sichuan together with three ex-situ cultivation populations from Yunnan and Hubei, China between September 2017 and October 2018. All the wild materials were collected outside at any natural reserves. All the cultivated materials were collected under the owner’s permission. In total, 364 individuals from 24 populations were sampled, 4–17 individuals were collected randomly in each population at intervals of 10 m. The collected plant issues were dried using silica gel. Detailed information about localities and samples are given in Table 1 and Fig. 5. Two voucher specimens were collected for each population and deposited in the herbarium of Kunming Institute of Botany, Chinese Academy of Sciences (code TR201701–TR201724).

### DNA extraction, primer selection, PCR procedure, and product detection

The genomic DNA was extracted from approximately 5 g of dried leaves of each collected sample using the modified CTAB method [48]. DNA concentrations were estimated by nano drop spectrophotometer (ND 2000, USA) and the quality was analyzed by electrophoresis on 2% agarose gel. Microsatellite markers were designed and synthesized on the base of Genome Skimming data obtained from sequencing by MiSeq Benchtop Sequencer (Illumina) using MISA software. Totally 180 pairs novel microsatellite markers were developed, from which 80 pairs microsatellite markers were selected to amplify and finally 13 pairs microsatellite markers were successfully amplified with high polymorphism (microsatellite markers information are shown in Table 2). The polymerase chain reactions (PCR) were carried out at a volume of 20 μL containing 50 ng template DNA, 0.5 μL of each primer, 10 μL 2 × Taq PCR MasterMix (Tiangen: 0.1 U Taq Polymerase/μL, 0.5 mM dNTP each, 20 mM Tris-HCl (pH 8.3), 100 mM KCl, 3 mM MgCl_2_). PCR amplification was performed under the following conditions: 95 °C for 3 min, 32 cycles of 95 °C for 30 s, annealing at 56–60 °C for 30 s, and elongation at 72 °C for 30 s, and a final extension step at 72 °C for 5 min. The PCR products were separated and visualized using the QIAxcel capillary gel electrophoresis system (QLAGEN, Irvine, California, USA).

### Data analysis

Data from QIAxcel capillary gel electrophoresis were analyzed by GeneMarker V. 2.2.0 to get allele fragment data. Micro-checker software was used to detect whether null alleles were present [30] and CERVUS software was used to calculate their frequency of each microsatellite loci [49]. Population genetic diversity parameters including average of sample sizes (*N*), average number of alleles (*Na*), effective number of alleles (*Ne*), Shannon’s information index (*I*), expected heterozygosity (*He*) and observed heterozygosity (*Ho*), fixation index (*F*) and percentage of polymorphic loci (*PPL*) were detected using GeneAlex version 6.0. *F*-statistics (*Fis*, *Fit* and *Fst*) were estimated for each locus across all populations using Fstat version 2.9.3.2. Genetic distances and genetic identity between each pair of accessions were measured from shared allele frequencies using PopGene 32. A dendrogram was constructed based on *Nei*’s genetic distance matrix using the MEGA version 4 software using the unweighted pair group method and the arithmetic averages (UPGMA) algorithm [50]. An analysis of the molecular variance (AMOVA) was used to verify the diversity within and among populations using Arlequin software version 3.5.1.3 [51]. A Mantel test [52] to compare pairwise geographic distance and pairwise genetic distance in terms of *Fst* / (1-*Fst*) with 1000 random permutations was conducted using NTSYSpc software version 2.10e [53]. The geographical distances among populations were calculated using the program Franson CoordTrans version 2.3. The population structure (the number of potentially different clusters) was assessed with a Bayesian-based cluster analysis using the program STRUCTURE version 2.3.4 [31]. Admixture model (AD) were tested with 10,000 replicates for burn-in and 10,000 replicates for Markov Chain Monte Carlo (MCMC) processes through five iterations (runs). To obtain the most probable K value (number of genetic groups), values of K from 1 to 24 were tested, with 10 independent runs for each K. The K value with the greatest probability was calculated estimating the maximum value of the ΔK statistic, according to Evanno et al. [54].

## Supplementary information

**Additional file 1: Table S1**. Paired *Nei*’s genetic distance (below diagonal) and genetic identity (above diagonal) of 24 populations of *Amorphophallus albus*

## Data Availability

All data generated or analysed during this study are included in this published article.

## Abbreviations

AD: Admixture model
AFLP: Amplified fragment length polymorphism
AMOVA: Analysis of molecular variance
CTAB: Cetyltriethylammnonium bromide
*F*: Fixation index
*Fis*: mean inbreeding coefficient within individuals relative to subpopulation
*Fit*: mean inbreeding coefficient within individuals relative to the total population
*F (null)*: Null allele frequency
*Fst*: mean inbreeding coefficient within subpopulation relative to the total population
*He*: The expected heterozygosity
*Ho*: The observed heterozygosity
*HW*: Hardy-Weinberg equilibrium
*I*: Shannon’s information index
ISSR: Inter-simple sequence repeat
KGM: Konjac glucomannan
MCMC: Markov Chain Monte Carlo
*N*: The average of sample sizes
*Na*: The average number of alleles
*Ne*: The effective number of alleles
*Nm*: Gene flow
PCR: The polymerase chain reactions
*PIC*: Polymorphism information content
Pop: Population
*PPL*: The percentage of polymorphic loci
PSESP: Plant species with extremely small populations
RAPD: Random amplification polymorphic DNA
RFLP: Restriction fragment length polymorphism
UPGMA: The unweighted pair-group method of arithmetic
